# Migration/Invasion of Malignant Gliomas and Implications for Therapeutic Treatment

**DOI:** 10.3390/ijms19041115

**Published:** 2018-04-08

**Authors:** Ching-Ann Liu, Chia-Yu Chang, Kuo-Wei Hsueh, Hong-Lin Su, Tzyy-Wen Chiou, Shinn-Zong Lin, Horng-Jyh Harn

**Affiliations:** 1 Bioinnovation Center, Buddhist Tzu Chi Medical Foundation, Hualien 97002, Taiwan; sagianne@gmail.com (C.-A.L.); Scata0726@gmail.com (C.-Y.C.); fskenneth16@gmail.com (K.-W.H.); Shinn-Zong@tzuchi.com.tw (S.-Z.L.); 2 Department of Medical Research, Buddhist Tzu Chi Hospital, Hualien 97002, Taiwan; 3 Department of Life Sciences, National Chung Hsing University, Taichung City 402, Taiwan; suhonglin@gmail.com; 4 Department of Life Science, National Dong Hwa University, Hualien 97002, Taiwan; twchiou@gms.ndhu.edu.tw; 5 Department of Neurosurgery, Buddhist Tzu Chi Hospital, Tzu Chi University, Hualien 97002, Taiwan; 6 Department of Pathology, Buddhist Tzu Chi Hospital, Tzu Chi University, Hualien 97002, Taiwan

**Keywords:** glioma, AXL/EZH2, epithelial-mesenchymal transition (EMT), phenotypic shift, cancer stem cells, microRNA, exosomes, invasion/metastasis

## Abstract

Malignant tumors of the central nervous system (CNS) are among cancers with the poorest prognosis, indicated by their association with tumors of high-level morbidity and mortality. Gliomas, the most common primary CNS tumors that arise from neuroglial stem or progenitor cells, have estimated annual incidence of 6.6 per 100,000 individuals in the USA, and 3.5 per 100,000 individuals in Taiwan. Tumor invasion and metastasis are the major contributors to the deaths in cancer patients. Therapeutic goals including cancer stem cells (CSC), phenotypic shifts, EZH2/AXL/TGF-β axis activation, miRNAs and exosomes are relevant to GBM metastasis to develop novel targeted therapeutics for GBM and other brain cancers. Herein, we highlight tumor metastasis in our understanding of gliomas, and illustrate novel exosome therapeutic approaches in glioma, thereby paving the way towards innovative therapies in neuro-oncology.

## 1. Introduction

Gliomas are primary tumors that arise from neuroglial stem or progenitor cells, which make up about 30% of all brain and central nervous system tumors, and 80% of all malignant brain tumors [1]. Gliomas are the most common primary intracranial tumors in adults [2], with half of all newly diagnosed gliomas classified as glioblastoma; 80% of GBMs arise de novo as primary GBMs. High grade gliomas are more common than low grades. Glioblastoma is associated with short survival and uniformly fatal outcome irrespective of treatment. Most tumors develop resistance to treatment and recur quickly due to heterogeneity. Following recurrence, glioblastoma is almost immediately fatal in the majority of cases. Glioblastoma is characterized by a high rate of local recurrence due to intrinsically radio-resistant, as well as chemo-resistant and tumor cell clones [3]. Originally, GBM was classified by histology according to WHO criteria: Grade I, pilocytic astrocytoma; Grade II, diffuse astrocytoma; Grade III, anaplastic astrocytoma; and Grade IV, glioblastoma [4]. The Cancer Genome Atlas (TGCA) Network recently catalogued recurrent genomic abnormalities in glioblastoma, and classified GBM into Proneural, Neural, Classical and Mesenchymal subtypes according to the molecular classification [5]. The major features of the Proneural subtype are point mutations in isocitrate dehydrogenase 1 (*IDH1*) and *TP53* mutation. *TP53* mutation accounts for 54% of the total cases of GBM. Among age-based statistics, the age of Proneural GBM patients was much younger than the other categories. Neural subtype is typified by the expression of neuron markers, such as neurofilament light (*NEFL*), γ-aminobutyric acid type A receptor alpha-1 subunit (*GABRA1*), synaptotagmin-1 (*SYT1*) and solute carrier family 12 member5 (*SLC12A5*). Patients with the Neural subtype are usually elderly people, and have no significant mutations. Classical GBM classification is based on the high rates of epidermal growth factor receptor (*EGFR*) alteration, and a distinct lack of tumor protein 53 (*TP53*) mutation. The *NF1* mutation was a proxy for the mesenchymal group, often accompanied by *PTEN* and *TP53* mutations, accounting for about 37% of patients with GBM [4,5] (Table 1).

The mutation frequency of IDH1 is extremely high and significant in the Proneural subtype of GBM (Table 1). IDH1 is one of three isocitrate dehydrogenase isozymes; it forms an asymmetric homodimer in the cytoplasm [7]. Wild-type IDH1 protein is found in the cytoplasm, peroxisomes and endoplasmic reticulum, and catalyzes the reversible oxidative decarboxylation of isocitrate to α-ketoglutarate (α-KG) as part of the TCA cycle in glucose metabolism [7,8,9,10]. Notably, IDH1 is the primary producer of NADPH in most tissues, especially in the brain [8]. Mutations in IDH1 associated with glioblastomas map to the highly conserved residue R132, resulting in an Arg (R) to His (H) substitution [11,12,13]. *IDH1* mutations result in a loss of normal enzymatic function and the abnormal production of 2-hydroxyglutarate (2-HG) [12]. 2-HG has been found to inhibit the enzymatic function of many α-KG dependent dioxygenases, including histone and DNA demethylases, causing widespread changes in histone and DNA methylation, and potentially promoting tumorigenesis [13]. Approximately 55–80% of secondary glioblastomas, those that progress from low-grade diffuse astrocytoma or anaplastic astrocytoma and occur in younger patients, have somatic mutations in the isocitrate dehydrogenase 1 (*IDH1*) gene, which are absent in primary glioblastoma in older patients [9,14,15,16,17]. Secondary glioblastomas have a significantly better prognosis than primary glioblastomas.

In general, patients with glioblastoma have a median survival of only 15 months [18,19]. Currently, the treatment of glioblastoma involves surgical removal of the tumor, followed by radiation with concurrent and adjuvant temozolomide [18]. Additional treatment options include passive and active immunotherapy, and the application of angiogenesis inhibitors in combination with chemotherapeutics and gene/antibody therapy. However, none of these therapies have been successful in curing GBM due to the presence of the blood–brain barrier (BBB), and the invasive nature of brain-tumor cells [20,21].

Malignant cancers spread in two phases. Firstly, tumor cells metastasize to another organ typically through haematogenous and lymphatic routes. The second phase involves local intra-organ invasion, whereby cells infiltrate an organ to form a new tumor. Distant metastasis of glioblastoma multiforme (GBM) most commonly occurs through cerebral-spinal fluid flow [22,23], but metastasis of GBM to the outside of the central nervous system (CNS) is rare [21,24,25,26]. Incidences of extraneural metastasis of GBM were reported at 0.2% [27]. Most of these cases had multiple metastases with primary site progression. In light of the overall poor outcome from current therapies, a better understanding of the unique biology of glioma invasion may provide brain-specific interventions for this rapidly progressing disease.

The mechanism of GBM invasion is an intricate program that takes place in the embryonic cells during development and in carcinoma cells during metastasis formation; so-called epithelial-to-mesenchymal transition (EMT). GBM cells undergo a series of molecular and conformational changes shifting the tumor towards mesenchymal traits, including extracellular matrix (ECM) remodeling, cytoskeletal re-patterning, and stem-like trait acquisition.

ECM remodeling has received much attention in the invasion of GBM because of its direct interaction with GBM cells in the tumor microenvironment. The extracellular matrix (ECM) of the tumor microenvironment plays a significant role in directing cellular behaviors, and serves as a passive scaffold in GBM growth and migration. The major component of brain ECM is polysaccharide hyaluronic acid (HA) [28,29]. HA is constitutively produced in GBM [30,31], its cellular receptor CD44 is also overexpressed in GBM, suggesting that CD44-enriched GBM cells are more efficient in invading the brain parenchyma [32,33]. Ligation of CD44 with HA activates the important pro-tumorigenic signals of Rho family of small GTPase [34,35], as well as PI3 kinase (PI3K) [36], which are known to affect cell motility, growth, proliferation and differentiation. This is in addition to collagen IV, collagen V, fibronectin and laminin, which in GBM-associated vessels, have been found to enhance cell survival, proliferation and migration in vivo and in vitro [37,38,39,40,41]. Attachment to ECM, including collagen, fibronectin and laminin, is controlled by the transmembrane receptors integrins. Concernedly, adhesion of integrins to their ligands significantly desensitize GBM cells to therapy [42]. For instance, α1 integrin signaling is negatively correlated with drug-induced apoptosis [43], and α6 integrin is necessary for the self-renewal, proliferation and tumor-forming capacity of GBM stem cells [40,44].

**Figure 1:**
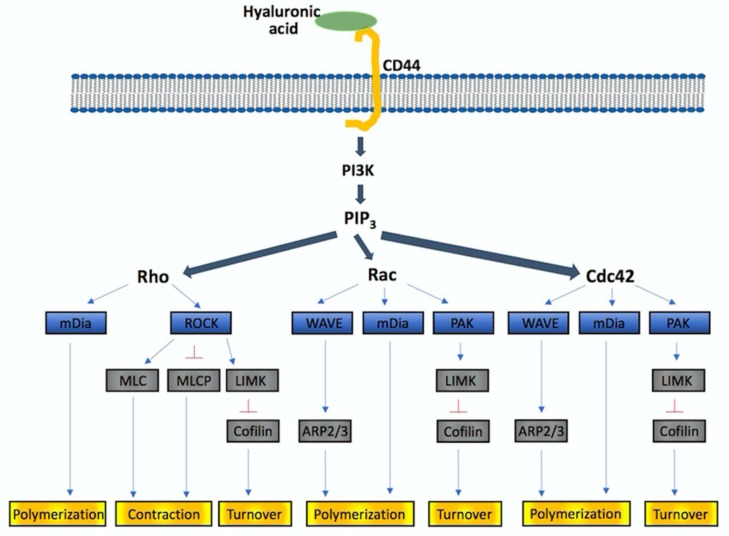
HA-CD44 triggers PI3K/Rho signaling for cytoskeletal re-pattering. Blue arrow (→): Promotion or activation; Red Y bar (⊥): Inhibition.

Malignant cancer cells utilize their intrinsic migratory ability to invade adjacent tissues and vasculature, and ultimately metastasize. Several studies revealed that molecules linking migratory signals to the actin cytoskeleton are upregulated in invasive and metastatic cancer cells. Cell migration is a highly integrated multistep process that is initiated by the protrusion of the cell membrane, termed filopodia, lamellipodia, and invadopodia/podosomes. Extracellular HA binds to the transmembrane receptor CD44 to trigger the PI3K and Rho GTPases. Rho GTPase signaling, which play role in regulating the polymerization of actin to produce stress fibers, filopodia and lamellipodia, coordinates control of cell motility and GBM invasion (Figure 1).

Ligation of CD44 with HA activates PI3K to convert PIP_2_ to PIP_3_, followed by activation of the Rho family of small GTPase, including Rho, Rac and Cdc42. The downstream targets of active Rho GTPases, with bound GTP, include kinases (p21-activated kinase, PAK; Rho-associated coiled-coil kinase; ROCK) and nucleation promoting factors (mammalian Diaphanous formin, mDia; Wiskott–Aldrich syndrome protein, WASP; Wiskott–Aldrich syndrome protein-family verprolin homologous protein, WAVE). While nucleation by mDia produces unbranched actin filaments, WASP and WAVE interact with the Arp2/3 complex and generate branched microfilaments. PAK phosphorylates LIM-motif containing kinase (LIMK), that in turn phosphorylates and inhibits cofilin, thereby regulating actin-filament turnover. Besides stimulating actin-filament growth, Rho GTPase promotes myosin actin interactions through ROCK. ROCK phosphorylates a number of actin cytoskeleton regulators including myosin II light chain (MLC), myosin light chain phosphatase (MLCP), and LIMK. Direct phosphorylation of MLC or MLCP has an immediate impact on the level of phosphorylated myosin light chain, which contributes to contractility. Rho mainly activates the formation of stress fibers and focal adhesions, Rac activates the formation of lamellipodia and membrane ruffles, and Cdc42 activates the formation of filopodia.

GBM poses several unique challenges to the currently available treatments. For example, GBM cells have the propensity to aggressively infiltrate/ invade the normal brain tissues and along the vascular tracks, which prevents complete resection of all malignant cells and limits the effect of localized radiotherapy while sparing normal tissue. Although anti-angiogenic treatment exerts anti-edematic effects in GBM, unfortunately, tumors progress with acquired increased invasiveness. Therefore, it is an important task to gain deeper understanding of the intrinsic and post-treatment invasive phenotypes of GBM in hopes that it will lead to novel GBM treatments that are more effective and less toxic. The urgency of diversified tumor cell subpopulations in malignant neoplasms contribute to the enhanced abilities to survive, grow, invade and metastasize (tumor progression). Phenotypic shifts and cancer stem cells play important roles in tumor progression, including tumor invasion and metastasis in GBM. Although a variety of signaling molecules contribute to the invasion and metastasis in GBM, we found notably that AXL/EZH2/TGF-β1 might be a key regulator in tumor invasion, migration, and EMT. The data demonstrated that the migratory and invasive capabilities of GBM stem-like cells can be reduced by suppressing expression of AXL/EZH2 by BP treatment. These results might help in the development of a new anticancer compound targeting the treatment of GBM. Accumulated evidences emphasize the roles of cancer-derived exosomes and miRNA in tumor migration, and indicate potential novel strategies for therapeutic drug development in GBM. Therefore, this review will give an overview of some of the signaling pathways that have been shown to participate in GBM invasion/metastasis, including the pivotal role of cancer stem cells (CSCs); phenotypic shifts, including (1) epithelial-mesenchymal transition (EMT) modulated by PI3K/AKT/mTOR (PAM) signaling; (2) proneural-mesenchymal shift; (3) migration/proliferation dichotomy; (4) angiogenesis-invasion shift; (5) Glial-Mesenchymal Transition (GMT); EZH2/AXL metastasis in tumor growth factor-β (TGF-β activation; microRNAs (miRNA)/drugs in inhibition of invasion and metastasis; and the role of exosomes in tumorigenesis, metastasis and therapeutic potentials. This review will also discuss the approaches to cancer therapies potentially altering GBM invasiveness.

## 2. Cancer Stem Cell Contribution to Glioblastoma Invasiveness

The brain gives rise to tumors with defined cellular hierarchies, suggesting that cancer replicates ontogenesis [45]. Radiation therapy (RT) is the standard curative treatment for a number of malignant tumors. However, radiotherapy has been found to promote the invasion of various kinds of cancer cells including GBM [46,47,48]. Evidence mounting in support of GBM recurrence and invasive nature are in part due to the presence of CD133^+^ cancer stem cells (CSC), which display radioresistant, chemoresistant, self-renewal, and tumorigenic potential [49,50,51]. Accumulating evidence indicates that proteins involved in the processes of migration and invasion, such as matrix metalloproteinases (MMPs), along with a disintegrin and metalloproteinases (ADAMs) and ADAM with thrombospondin motifs (ADAMTS) families, are upregulated in GBM CSCs [52,53,54,55,56]. Moreover, putative stem cell markers such as L1 cell adhesion molecule (L1CAM), nucleostemin, and nestin have been found at the leading edge of the tumor [57,58,59], supporting opinions that CSCs are responsible for GBM invasion. Brabletz et al. proposed that CSCs in situ can transform to migrating stem cells by EMT, the migrating stem cells then disseminate and form metastatic colonies [60]. Recently, the novel role of SOX2 as a key molecule in the invasive and migration properties of GBM CSCs and glioma cell lines has drawn attention to cancer research [61]. The *SOX2* gene encodes a transcription factor containing a high mobility group (HMG)DNA-binding domain [62]. SOX2 expression is limited to stem cells and progenitor cells [63]. A mouse model revealed that SOX2 is required for the maintenance of CSCs in high grade oligodendroglioma [64], thus, affirming the potential importance for SOX2 in brain neoplasm tumorigenicity. However, the role of SOX2 in GBM invasion is still unclear.

## 3. Phenotypic Shift in GBM

GBM cells have the ability of phenotypic shifts that are conducing to their proliferation, angiogenesis and invasion [65,66]. The mechanisms that correlate to phenotypic shift are epithelial-mesenchymal transition (EMT), proneural-mesenchymal shift, migration/proliferation dichotomy (Go or Grow mechanism), angiogenesis-invasion shift [66] and glial-mesenchymal transition [67].

### 3.1. Epithelial-Mesenchymal Transition (EMT) and Metastasis

**Figure 2:**
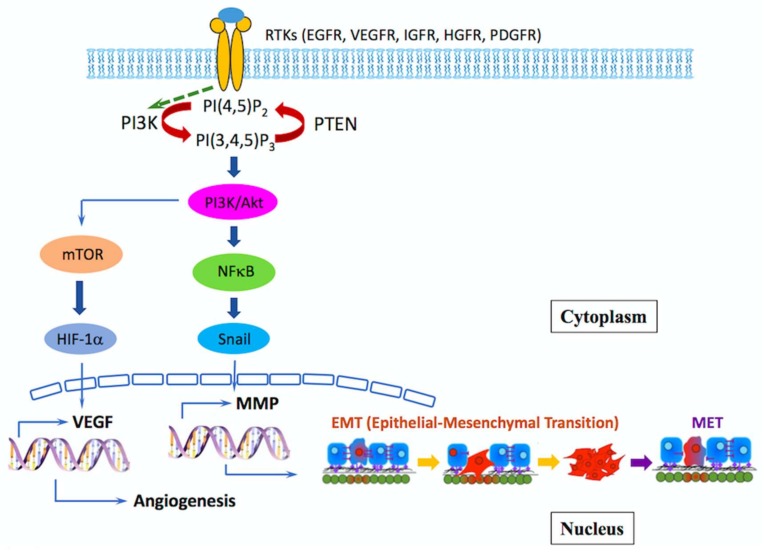
PI3K/Akt/mTOR (PAM) signaling network and effector functions associated with metastasis.

Cancer metastasis is the major cause of cancer morbidity and mortality that accounts for approximately 90% of cancer deaths. The highly invasive nature of GBM impedes the surgical removal of all tumor cells, making relapse inevitable. However, the real relevance of this program in malignant glioma is still controversial, and the mechanisms used by glioma cells to invade the surrounding tissue are still unclear. H. A. Fine et al. demonstrated that invading glioma cells (IGCs) were found to have reduced expression of genes within the extracellular matrix compartment, and genes involved in cell adhesion, cell polarity and epithelial to mesenchymal transition (EMT) processes [68]. EMT is a biological process that allows immobile epithelial cells to acquire a mobile mesenchymal phenotype, becoming detached and invasive. It was initially described in the context of embryonic differentiation [69]. In tumor cells, EMT, together with the induction of neo-angiogenesis, initiates cancer metastasis, inducing enhanced migratory properties, invasiveness and resistance to apoptosis [70,71]. Recent experimental and clinical studies have also implicated EMT and its reverse program, mesenchymal–epithelial transition (MET), in the metastatic process (Figure 2), and are strongly associated with GBM malignancy [71,72].

PAM-signaling network and effector functions associated with metastasis: In GB and MB, aberrant PAM signaling can promote tumor progression by over- inducing angiogenesis, EMT, cell migration and invasion, and also by inhibiting loss of adhesion associated apoptosis. As a tumor cell undergoes EMT, it begins to lose its epithelial phenotype. Loss of cell-to-cell attachment receptors and integrins (shown in purple) also occurs and beyond. Once a cancer cell has completely undergone EMT and travels to a new location, multiple steps (not explicitly shown) involving MET must occur for the metastatic cancer cell to anchor to the distant site and form a secondary tumor.

During EMT, a variety of transcription factors are upregulated in metastatic cells, such as Snail, Slug, Twist and Zeb 1/2. [73]. Twist overexpression has also been correlated with the induction of tumor cell invasion in GBM [74]. However, these malignancies usually do not metastasize out of the GBM, mainly due to their rapid relapse rate and poor prognosis [75]. Even so, there are reports describing GBM metastasis involving the spread of GBM cells out of the CNS through cerebrospinal fluid, blood or lymphatic vessels [76,77]. Medulloblastoma (MB), on the other hand, has a high tendency to disseminate to the spinal cord and leptomeninges of the cerebellum and forebrain. The PI3K/AKT pathway is activated in 50% of GBMs. In the case of MB, there are a number of studies concerning alterations in this pathway [78,79,80]. This pathway appears to facilitate an invasive phenotype of GBM and MB, especially in terms of motility and resistance to stress [81].

During the EMT process, malignant cells start to intravasate into the surrounding blood vessels in order to migrate to other parts of the body. To accomplish this process, the extracellular matrix and basement membrane of blood vessels have to be degraded by matrix metalloproteases (MMP) [82]. The most relevant metalloproteases in this invasive process are MMP-2 and MMP-9 [83]. One of the upstream pathways controlling MMP production is the PI3K/AKT pathway [84]. Thus, drugs like wortmannin, which inhibits the secretion of MMP-2, block GBM invasion through the down-regulation of the PI3K/AKT/NF-κB signaling pathway [85]. Since Snail induces MMP-9 expression, EMT seems to be necessary for intravasation of lymph vessels in GBM and other cancers [86]. Greenspoon, JN et al. also demonstrated that EMT has been shown to cooperate with MMP activity in glioblastoma multiform (GBM), allowing cells to gain access to lymph vessels. Preliminary data suggest this new EMT-associated drug target, in combination with stereotactic radiosurgery, may provide potential targets for future treatment [87].

Upstream regulators of EMT induction, such as insulin-like growth Factor-1 receptor (IGF-1R), c-MET and the CXCR4 receptor, have been proposed as potential targets to inhibit GBM or MB invasion. IGF-1R is typically overexpressed in malignant GBM [88], and its activation by IGF-1 contributes to Snail and Twist expression though PI3K/AKT signaling pathway activation [89,90]. Therefore, IGF-1R tyrosine kinase inhibitors or IGF-1 inhibitors, such as osthole, have been used to inhibit GBM proliferation, migration and EMT [90,91].

c-MET expression levels correlate with tumor grade in CNS malignancies [92], and its activation also mediates EMT-promoting signals in cancer cells via class IA PI3K. The use of c-MET kinase inhibitors, such as SGX523, suppresses tumor growth in GBM cell lines [93]. This inhibition blocks the EMT induced by VEGF ablation in a GBM mouse model [94] and induces an effective decrease in MB cell migration and invasion [95].

Stromal cell derived factor (SDF-1) or CXCL2 and its chemokine receptor CXCR4 can induce EMT in GBM via activation of PI3K/AKT and extracellular-signal-regulated kinase (ERK) pathways, and its inhibition suppresses EMT in glioma cell lines by upregulating E-cadherin [96]. However, single agents targeting the PAM (PI3K/AKT/mTOR) pathway have been reported to be an inefficient approach in MB and increase invasion in the surviving fraction of GBM [97]. Therefore, new therapeutic approaches should be based on increasing the therapeutic window by targeting two different routes, namely the PAM and ERK pathways, or on combining PAM inhibitors with chemotherapeutic agents [98].

**Figure 3:**
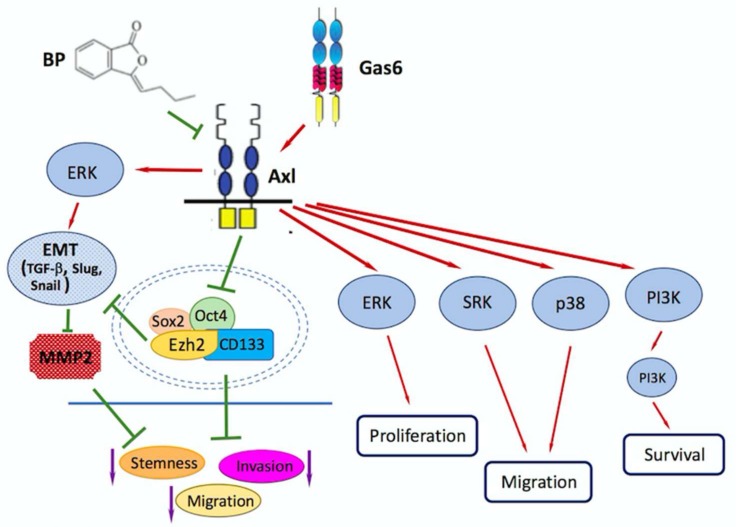
The schematic mechanism of BP treatment and Gas6 activation in GBM cells.

Our previous study also demonstrated that Ezh2 over-expression recovers the EMT-associated markers TGF-β, Slug and Snail, which then activate MMP2 to participate in promoting GBM invasion/ migration and maintaining stemness of GBM cells (Figure 3).

BP treatment reduces expression of Axl and stemness genes, including Ezh2, sequentially inhibits migration/invasion of GBM. BP-mediated Axl/Ezh2 suppression participates in TGF-β related EMT also contributes to inhibition in GBM metastasis. Nevertheless, Gas6/Axl signaling activates downstream molecules, including ERK1/2, SRK, p38 and PI3K/Akt, promotes cell proliferation, migration and survival in GBM.

The capacity of cancer cells to survive and invade depends, at least partially, on the acquisition of mesenchymal characteristics through EMT [99]. However, the capacity of tumor cells to form metastasis depends on their ability to revert back to an epithelial phenotype known as mesenchymal-to-epithelial transition (MET), and it is crucial to the conclusion of metastasis [100,101]. The investigations of EMT in GBM are relatively recent. Therefore, only a limited amount of information is available about the differences in the classical EMT that occurs in epithelial tumors and the glial–mesenchymal changes that occur in high-grade gliomas [99].

According to Paget (1989), who first pointed out the “seed and soil” theory, tumor metastasis is dependent on local properties that are inherent to the environment where the circulating tumor cells (CTCs) anchor [102]. Once cancer cells reach the circulation, the bloodstream or lymphatic system, EMT will drive epithelial cells to avoid anoikis, then MET, the reverse process of EMT, will govern occurrence of metastasis in the target tissue [103,104]. Since glioma is not a tumor of epithelial origin, the mesenchymal molecular and cellular changes occur in an independent way of a “cadherin switch” in GBM [99]. This can be explained mainly because repressors of E-cadherin are able to also regulate other invasion and migratory-associated genes.

### 3.2. Proneural-Mesenchymal Shift

Radiotherapy is the standard treatment of GBM following surgery resection. Radiation has been demonstrated to induce a phenotypic shift from a proneural pattern toward the mesenchymal one in malignant gliomas at the time of tumor recurrence [105]. Radiation-induced proneural to mesenchymal shift was approached using an in vivo glioma model [106]. In this study, the radiation responsive regulators p53 and e2F, targets of Stat3 and ceBPB, were up-regulated. IL-15, LIF, and IL-7 (activators of Stat3 via gp130/Jak) are also up-regulated following radiation. The results suggest that cytokine-mediated activation of JAK/STAT pathway may drive the proneural to mesenchymal shift. Additionally, microglia-derived TNF-α induces mesenchymal to proneural GBM neurospheres via NF-κB activation [107]. Both of NFkB and JAK/STAT are mechanisms of EMT; and the EMT and proneural-mesenchymal shift lead to increased invasion of glioma cells, whether the proneural-mesenchymal shift and EMT are the same, or there is crosstalk between these two events remains to be elucidated.

### 3.3. Migration/Proliferation Dichotomy (Go or Grow Mechanism)

GBM progression is determined by cell proliferation rate and migration speed. Experiments with cultured glioma cells observed that tumor cells harvested from the vital core of a GBM rapidly grew to large colonies in soft agar, whereas cells from regions of invaded brain developed smaller colonies [108]. On the contrary, migration assay showed higher motility of invaded brain compared to cells from solid tumor. These results suggest that invasive glioma cells are more likely migratory than proliferative, which is known as the migration/proliferation dichotomy or ‘Go or Grow’ mechanism [109]. The Go or Grow transition can be induced by stress stimuli, such as hypoxia or irradiation.

### 3.4. Angiogenesis-Invasion Shift

In rapidly growing GBM tumors, the oxygen and glucose may fluctuate. Therefore, glioma cells ensure an adequate oxygen and glucose supply through increased angiogenesis or migration. Bikfalvi identified the angiogenesis-invasion shift in an experimental glioma model, and the results indicate that anti-angiogenesis treatment drives the expression of critical genes which relate to disease aggressiveness in GBM patients [110]. Immunohistochemical analysis of human GBM samples confirmed higher expression levels of annexin A2 in tumor cells clustered around neovasculatures, but not in diffusely invasive tumor cells.

### 3.5. Glial-Mesenchymal Transition (GMT)

Glioblastoma is characterized by a high rate of local recurrence due to intrinsically radio-resistant or chemo-resistant tumor cell clones. It is known that EMT is involved in irradiation-induced cancer progression. Tanino M et al. raise a novel concept of glial-mesenchymal transition (GMT) [67]. GMT is an irradiation-induced mesenchymal phenotype in malignant gliomas (MGs). Irradiation triggers the ERK1/2 and GSK3β phosphorylation, together with the microenvironment-mediated TGF-β-induced phosphorylation of Smad2/3, leading to the elevation of Snail expression in MGs. Sustained elevation of Snail, which plays an important role in migration and invasion by upregulating expression of vimentin, CD44, collagen and αSMA (α-smooth Muscle actin) in MGs, may contribute the phenotypic shift through ERK1/2, GSK3β and TGF-β pathway.

## 4. Role of EZH2/AXL/TGF-β axis in Glioblastoma Metastasis/Invasion

The majority of deaths (about 90%) associated with cancer are due to metastasis; 25% of patients who die from cancer have CNS metastases detected at autopsy. Of these, about 15% are in the brain and in about 10%, the brain is the only site of CNS metastases. In the recent years, the enhancer of Zeste 2 (EZH2) is considered to be a therapeutic target in cancer research [111]. Enhancer of zeste homolog 2 (EZH2) is the catalytic subunit of the Polycomb-repressive complex 2 (PRC2) that epigenetically silences gene transcription through histone H3 lysine trimethylation (H3K27me3) during development and cellular differentiation [112]. Studies on human tumors show that *EZH2* gene is highly similar in organisms ranging from flies to humans. EZH2 is over-expressed in different cancer types, including hematological and solid malignancies, as well as malignant glioma [113], which is thought to promote tumor progression by silencing tumor suppressor genes [114,115,116]. EZH2 upregulation in gliomas maintains stemness of tumor cells by inhibiting their differentiation [117,118]. The therapeutic approaches have indicated that EZH2 controls diverse phenotypic features of cancer including proliferation, invasiveness, metastasis and resistance to cell death [119,120,121]. Pre-clinical studies showed that EZH2 is able to silence several anti-metastatic genes (E-cadherin and tissue inhibitors of metalloproteinases), thereby favoring cell invasion and anchorage-independent growth [122]. In addition, EZH2 seems to play a crucial role in favoring tumor angiogenesis. EZH2 is able to inhibit the expression of DAB2IP, which codes for a Ras-GTPase-activating protein, leading to increased metastatic potential through Ras- and NF-κB-dependent pathway activation. Taken together, EZH2 may participate in tumor invasion/metastasis.

Michael Platten’s lab identified that AXL, a novel target gene in glioblastoma, is positively regulated by EZH2 and mediates invasiveness driven by EZH2 [123]. AXL is a multifunctional receptor tyrosine kinase implicated in neural and mesenchymal development that has been shown to mediate cell survival, proliferation, migration, invasion, and adhesion in multiple tumors [124,125,126,127,128,129], including glioma [130,131,132]. The AXL overexpression in GBM is thought to contribute to invasiveness, chemoresistance, and poor survival [133]. Axl/Gas6 signaling regulates proliferation, survival and differentiation in stem cells [134,135], which also protects axon integrity [136].

High expression of TGF-β has been reported in glioma tumor specimens and cell lines [137]. Rao et al. [8] found that EZH2 knockdown reduces expression of transforming growth factor β1 (TGFβ1), and increases E-cadherin expression. Furthermore, they observed a positive correlation between overexpression of EZH2 and TGFβ1 in ovarian carcinoma tissues [138]. Previous studies have shown that this pathway is involved in glioblastoma aggressiveness [139]. Transforming growth factor-β (TGF-β) represents a member of a large family of cytokines that include the bone morphogenic protein (BMPs), nodals and activins, which are involved in the regulation of embryonic development and tissue homeostasis [140]. Malignant gliomas are characterized by a high rate of invasiveness. Around 90% of all GBMs relapse at the surgical margins in close proximity to the resection cavity and only a small fraction of GBM (5–10%) recur at a greater distance from the main tumor mass [141]. The invasive phenotype of malignant gliomas has been associated with the activation of several cell surface receptors including receptor tyrosine kinases (RTKs), G protein-coupled receptors (GPCRs), tumor growth factor-β receptor (TGF-β receptor), integrins, immunoglobulins, tumor necrosis factor (TNF) family, cytokine receptors and protein tyrosine phosphatase receptors [142].

The correlation of Ezh2/Axl/TGF-β in glioblastoma migration/invasion has been researched in our previous study. The small molecule *n*-Butylidenephthalide (BP) reduced expression of AXL and stemness-related genes, including CD133, Sox2 and Oct4, in a dose-dependent manner in glioblastoma. Nevertheless, the mechanism of BP reducing expression of AXL and the stemness-related gens is still unknown, and we are approaching the vitally important issue of elucidating it in detail. AXL/EZH2 reduces migratory and invasive capabilities of GBM stem-like cells. Furthermore, our studies represent that AXL/EZH2/TGF-β1, but not Sox2, might play a pivotal role in tumor invasion, migration and EMT (Figure 2) [143]. The full understanding of the gene regulation of AXL-EZH2-TGF-β1 axis in GBM invasion, migration and EMT process is still limited, and further studies are close to elucidating the detailed mechanism.

BP treatment reduces expression of Axl and stemness genes, including Ezh2, sequentially inhibits migration/invasion of GBM. BP-mediated Axl/Ezh2 suppression participates in TGF-β related EMT, contributing to inhibition in GBM metastasis. Nevertheless, Gas6/Axl signaling activates downstream molecules, including ERK1/2, SRK, p38 and PI3K/Akt, promotes cell proliferation, migration and survival in GBM.

## 5. miRNAs/Drugs in Inhibition of Invasion and Metastasis

MicroRNAs (miRNAs) are small (19–25 nucleotide), noncoding RNA molecules that negatively regulate gene expression by base pairing with 3′-untranslated regions (3′-UTRs) of target mRNAs, leading to translational suppression or degradation [144]. Accumulated evidence suggests that miRNAs emerge as important players in tumorigenesis, acting as oncogenes or tumor suppressors depending on their cancer-related target genes [145]. A previous study has demonstrated that EpCAM regulates the miR-17-92 cluster expression that further influences cell proliferation and invasion in retinoblastoma cells; antagomirs significantly decreased cell viability and induced cell apoptosis [146]. Interestingly, miR-17/20a, the members of the miR-17-92 cluster, represented a negative correlation with TNM and lymphatic metastasis clinically [147]. Taken together, these results indicated that the same miRNA may play opposite roles in different cancers, therefore, discovery of miRNA targets and their relevant pathways is urgently needed. The formation of a metastasis reflects a succession of complex steps leading to the macroscopic outgrowth of disseminated tumor cells at the secondary site. Recently, certain microRNAs (miRNAs) have been shown to regulate either a single step or multiple steps of metastasis [148].

### miRNAs Regulate EMT in GBM

**Figure 4:**
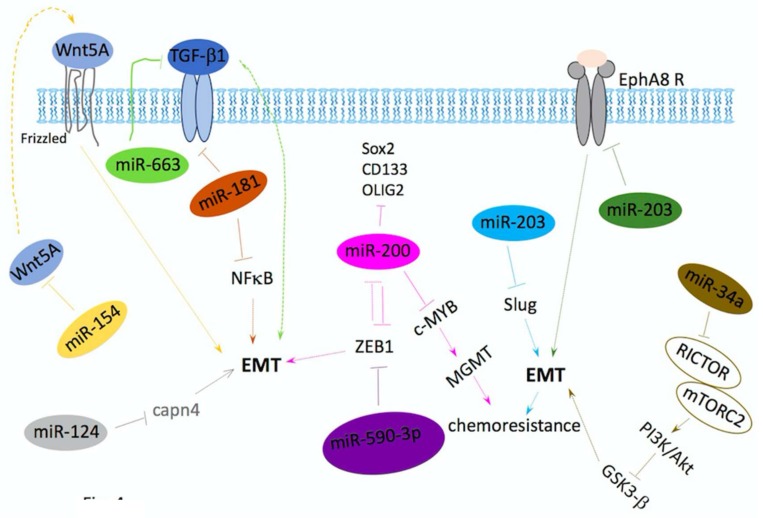
miRNAs regulate GBM invasion and progression through EMT. Solid arrows, activation; dotted arrows, putative activation; solid Y bar, inhibition; dotted Y bar, putative inhibition.

It has been reported that miRNAs play a critical role in cancer as well as in EMT in recent studies. These miRNAs dynamically impact the regulation of the epithelial–mesenchymal state by targeting cancer-related pathways, such as the proteins involved in invasion, migration, proliferation, DNA damage response and steaminess. miRNAs drive epithelial/mesenchymal plasticity and promote or inhibit the spreading ability of cancer cells [100,149,150]. miRNAs regulate GBM invasion and progression through EMT (summarized in Figure 4).

miRNAs can repress EMT by targeting ZEB (miR-590-3p, miR200), Slug (miR-203), Wnt5a (miR-154), TGFβ (miR-663), TGFβ receptor and NF-κB (miR-181), Rictor (miR34a), and Capn4 (miR-124), whereas miR10a can induce EMT by repressing the expression of the tyrosine kinase receptor EphA8, respectively. EMT-inducing genes can also regulate the expression of miRNAs, such as miR-200, in a double-negative feedback mechanism. This miRNA has impact on chemoresistance and stemness. EMT, epithelial–mesenchymal transition; TGFβR, transforming growth factor receptor; MGMT, *O*-6-methylguanine-DNA methyltransferase; RICTOR, rapamycin-insensitive companion of mammalian target of rapamycin; mTOR, mammalian target of rapamycin; Capn4, calpain small subunit 1.

miRNAs can repress EMT by targeting ZEB (miR-590-3p, miR200), Slug (miR-203), Wnt5a (miR-154), TGFβ (miR-663), TGFβ receptor and NF-κB (miR-181), Rictor (miR34a), and Capn4 (miR-124), whereas miR10a can induce EMT by repressing the expression of the tyrosine kinase receptor EphA8, respectively. EMT-inducing genes can also regulate the expression of miRNAs, such as miR-200, in a double-negative feedback mechanism. This miRNA has impact on chemoresistance and stemness. EMT, epithelial–mesenchymal transition, TGFβR, transforming growth factor receptor, MGMT, *O*-6-methylguanine-DNA methyltransferase, RICTOR, rapamycin-insensitive companion of mammalian target of rapamycin, mTOR, mammalian target of rapamycin and Capn4, calpain small subunit 1.

TGF-β pathway is targeted by miR-663 which is downregulated in GBM; miR-663 overexpression contributes to the inhibition of migration, invasion and proliferation in GBM [151]. miRNA 200 family targets ZEBs and regulates negative tumor development and EMT, whole-genome miRNA analysis of GBM shows that loss of miRNA200c expression is related with EGF receptor (EGFR) amplification and ZEB1 overexpression [152,153,154], suggesting the involvement of ZEB1 in tumor aggressiveness [155]. miR-590-3p, the miRNA also targets ZEB gene, whose expression levels are down-regulated in both GBM tissue samples and GBM cell lines. Overexpression of miR-590-3p will inhibit the motility, invasion and expression of epithelial gene in glioma cell lines [156]. miR-181 family and miR-154 also act as GBM suppressors. Expression of miR-181 or miR-154 is associated with poor prognosis in GBM patients. Besides, the expression of these two miRNAs reduced migration and invasion of GBM cell lines, and induction of MET. The MET induction by miR-181 or miR-154 is based on inhibitory effect on TGF-β, NF-κB and Wnt signaling respectively [157,158]. miR-124 expression is inversely correlated with EMT. The overexpression of miR-124 leads to inhibition of calpain small subunit 1 (Capn4), a protein that has been correlated with the invasion of several types of tumors, besides decreased expression of p-FAK, MMP2, vimentin, and N-cadherin, as well as impaired cell migration and invasion this cancer cells [159]. miRNA-203 is important in carcinogenesis. In GBM, miRNA- 203 targets Slug and inhibits EMT, decreasing the resistance to chemotherapy. miR-203 was significantly downregulated in the imatinib-resistant GBM cells. miR-203 was also downregulated in samples from patients diagnosed with GBM grade III/IV, when compared with grade I/II samples. Also, there is an inverse correlation between miR-203 expression level and Slug mRNA level in human GBM specimens [160].

miRNAs that are critical for tumor formation, maintenance, and progression might serve as targets for therapeutic intervention. Systemically delivered microRNA mimics inhibited tumor growth may be considered as a therapeutic strategy [161]. On the other hand, miRNAs that promote tumor formation and/or metastasis can be targeted in vivo. Therapeutic silencing of miR-10b using “antagomirs”, the chemically modified, cholesterol-conjugated antisense miRNA inhibitors, resulted in sequence-specific inhibition of metastasis in a mouse mammary tumor model [162]. miRNA-based agents have been shown to modulate sensitivity to traditional cancer drugs. Therefore, combination treatment with miRNA-based agents and other therapeutic drugs could be beneficial and may be innovative therapies in neuro-oncology.

## 6. Exosomes in Glioma Progression and Therapeutic Potentials

Extracellular vesicles (EVs) are known mediators of intercellular communication for both normal and tumor cells. Exosomes are one type of EVs which facilitate this intercellular communication and cross-talk within the tumor microenvironment. Exosomes secreted by tumor cells are increasingly recognized in a number of processes underlying tumor progression including facilitating the transport of receptors, signaling molecules, oncogenic genes and miRNA. They are emerging as a key component in the biogenesis of glioma, in addition to contributing to the modification of the surrounding microenvironment to support tumor progression.

Exosomes are small, lipid bilayer membrane vesicles of endocytic origin. Exosomes can be defined via a number of main morphological and physical characteristics. They range in size between 40 and 120 nm in diameter, are of endocytic origin and sediment at approximately 100,000 g (sucrose density gradient of 1.13–1.19 g/mL) [163]. Morphologically, they appear as spherical structures (“cup” or “dish” shaped in transmission electron microscopy) with a well-defined lipid bilayer when viewed with an electron microscope [164]. Contained within their aqueous core or in the lipid membrane, are various proteins, nucleic acids and receptors that are reflective of the parental donor cell. The variety of proteins and receptors that are present in exosomes is largely dependent on their cell of origin. Whilst the biogenesis of exosomes and cargo regulation is complex, it knows that exosomes are generated first as intraluminal vesicles (ILVs) within multivesicular bodies (MVBs), via mechanisms that are either dependent or independent of the ‘Endosomal Sorting Complex for Transport’ (ESCRT) [165]. The formation of exosomes has been shown to be controlled by the syndecan heparin sulfate proteoglycans and their cytoplasmic adaptor syntenin [166]. Accumulating evidence indicates that exosomes play important roles in cancer. Exosome cargos contained oncogenic proteins and nucleic acids play decisive roles in tumorigenesis, growth, progression, metastasis, and drug resistance.

## 7. Role of Exosome in Tumors

### 7.1. Tumorigenesis

**Figure 5:**
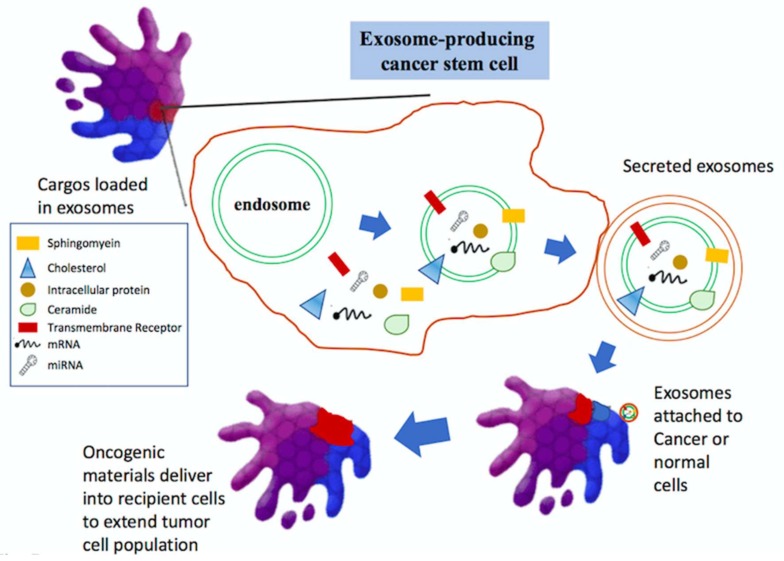
Roles of exosomes in cancer.

Exosomes from malignant cells have shown the potential to induce normal cell transformation. For instance, prostate cancer cell-derived exosomes could induce neoplastic transformation of adipose-derived stem cells (ASCs) which is associated with trafficking of oncogenic proteins, mRNA and miRNAs [167]. Melo et al. suggest that cancer cell-derived exosomes contain precursor microRNAs (pre-miRNAs) associated with RNA-induced silencing complex (RISC)-loading complex proteins, induce the rapid and efficient silencing of mRNAs in non-tumorigenic epithelial cells, resulting in transcriptome reprogramming and oncogenic transformation [168]. Weissleder and their laboratories analyzed the exosomal miRNAs participating in drug resistance in GBM. Microfluidic chip analysis has shown that mRNA levels of O^6^-methylguanine DNA methyltransferase (MGMT) and alkylpurine-DNA-N-glycosylase (APNG), the pivotal enzymes are able to repair tamozolozide-induced DNA damage and were increased in exosomes isolated from GBM [169]. Exosomes isolated from serum specimens from cancer patients induce tumor formation in mice when co-injected with the non-tumorigenic epithelial cells, suggesting a potential mechanism for exosome in tumorigenesis [168]. These findings indicate that exosomes may contribute to tumor progression by mediating the transformation of normal cells to malignant cells, and modulate the balance between cancer stem cells (CSCs) and non-CSCs (Figure 5).

Exosomes are critically involved in tumor initiation, growth, progression, metastasis, and drug resistance by transferring oncogenic proteins and nucleic acids.

### 7.2. Tumor Metastasis

Exosomes contribute to tumor metastasis by enhancing tumor cell migration and invasion, establishing pre-metastatic niche, and remodeling the extracellular matrix. The formation of pre-metastatic niche is a prerequisite for tumor metastasis. Tumor-derived exosomes can activate endothelial cells to support tumor angiogenesis and thrombosis. Tumor-derived exosomes can convert fibroblasts and mesenchymal stem cells (MSCs) into myofibroblasts to facilitate tumor angiogenesis and metastasis. Tumor-derived exosomes also contribute to immune response creating an immunosuppressive microenvironment by inducing apoptosis and impairing the function of effector T cells and natural killer cells (NK cells), inhibiting dendritic cells (DCs) differentiation, expanding myeloid-derived suppressor cells (MDSCs), as well as promoting regulatory T cell (Treg) activity. In turn, exosomes from activated T cells, macrophages, and stromal cells can promote tumor metastasis and drug resistance. Suetsugu et al. showed that highly metastatic breast cancer cells can transfer their own exosomes to other cancer cells and normal lung tissue cells in vitro and in vivo by using fluorescent protein imaging methods [170]. Glioma-derived extracellular vesicles (EVs) were shown to contribute to tumor invasion and metastases providing essential stimulatory signals to irradiated glioma cells. The connective tissue growth factor (CTGF) mRNA and insulin-like growth factor binding protein 2 (IGFBP2) protein levels were elevated in exosomes of irradiated gliomas; additionally, these exosomes promote the activation of molecules involved in cell migration, such as neurotrophic tyrosine kinase receptor type 1 (TrkA), focal adhesion kinase (FAK), paxillin, and proto-oncogene tyrosine-protein kinase Src (Src), in exosome-recipient cells [171]. The full-length neural adhesion/recognition protein L1 (L1CAM) is presented in exosomes produced by glioma cells [172]. A disintegrin and metalloproteinase domain-containing protein 10 (ADAM10), the matrix proteinase increases consistently with glioma grade, catalyzes proteolysis of L1CAM and releases the ectodomain of L1CAM [173]. The ectodomain interacts with FGF receptor, β1- and α5-integrins on the surface of recipient cells to activate integrin-linked kinase (INK) and FAK, and then induce cell motility through upregulation of PI3K/Akt and NF-κB [173,174,175,176]. Furthermore, glioma-derived EVs support tumor expansion and invasion by inducing apoptosis in normal brain cells surrounding the tumor [177]. Taken together, these findings reveal that the intercellular communication mediated by exosomes may be an important mechanism for tumor metastasis.

### 7.3. Exosomes Application as Anti-Cancer Drug Delivery Vehicles

**Figure 6:**
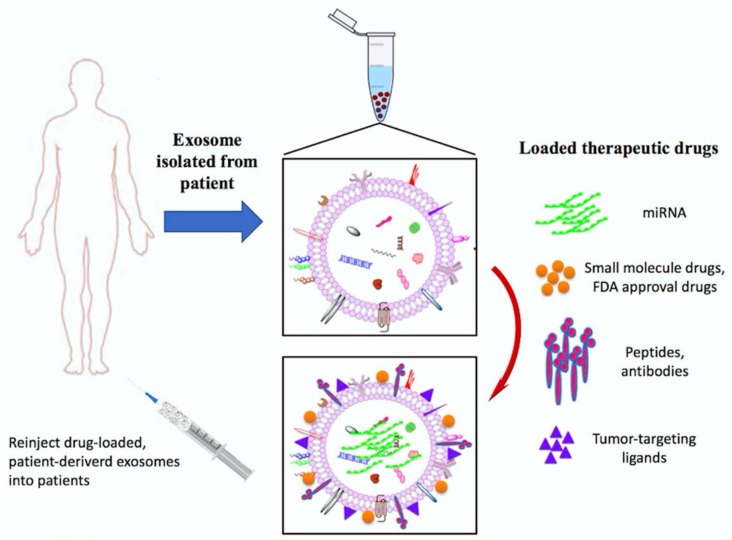
Exosomes as anti-cancer drug delivery vehicles.

The use of exosomes to deliver nucleic acids or drugs has gained considerable interest due to the excellent biodistribution and biocompatibility [178]. Exosomes are modified with targeting ligands, such as RGD, to improve drug delivery efficacy to tumors. The modified exosomes show highly efficient targeting to αV integrin-positive breast cancer cells, and intravenous injection of these exosomes obviously inhibits tumor growth [179]. In addition, exosomes from MSCs have been tested as the vehicle to package and deliver active drugs [180,181] (Figure 6). Munoz et al., delivers anti-miR-9 using the mesenchymal stem cell-derived (MSC) exosomes for GBM treatment [182]. The results demonstrated the chemo-resistance of GBM was reversed by exosome-delivered anti-miR-9. miR-124a was screened and identified as the efficient agent against glioblastoma. miR-124a delivered by MSCs-isolated exosomes resulted in dramatic reduction in cell viability and increase in survival rate in vitro and in vivo [183]. Glioma EVs carry the oncogenic antigens, as well as miRNAs, mRNAs and DNAs, that could serve as potential targets for the development of immune vaccines to induce or improve immune response specifically targeted gliomas [177]. All this evidence implicates that exosomes may be a potentially effective therapeutic for drug delivery in GBM treatment.

Peripheral blood samples are taken from cancer patients, exosomes are isolated, characterized and selected using exosome-specific markers, such as CD9, CD63, CD81 or Hsp70. followed by quantification by protein assays. Exosomes are infused into the same patient after preconditioning with chemotherapy.

## 8. Summary and Future Prospects

Tumor biology has developed over decades and significant improvements to cancer therapies have increased patients’ life spans. Gliomas notoriously develop drug resistance and/or upregulate compensatory pathways in response to monotherapies. Therefore, a better understanding of the metastasis relevance, signaling of glioma, and the associated use of these agents as adjuvants or as a part of a drug cocktail may meet greater clinical success.

The vast majority of current clinical trials approach gliomas from a traditional oncological perspective, focusing on the tumor per se. For a complete change in our treatment of gliomas to occur, novel aspects of glioma biology must be targeted. Elucidating the role of CSCs and the signaling pathways in cancer cells may direct research towards novel and specific agents that target CSCs (or these pathway proteins) to prevent any recurrence. In addition, we highlighted the crucial miRNAs, a class of short, non-coding ribonucleic acid molecules, as well as cancer-derived exosomes, influenced tumor metastasis, invasion and radio-resistance, signaling pathways that miRNAs and exosomes regulate GBM migration/invasion will hopefully open a new field of treatment strategies for glioblastoma treatment.

## Figures and Tables

**Table 1:** Frequently-mutated genes in GBM.

Gene	Proneural (*n* = 37)	Neural (*n* = 19)	Classical (*n* = 22)	Mesenchymal (*n* = 38)
*TP53*	20 (54%) *	4 (21%)	0 (0%)	12 (32%)
*PTEN*	6 (16%)	4 (21%)	5 (23%)	12 (32%)
*NF1*	2 (5%)	3 (16%)	1 (5%)	14 (37%) *
*EGFR*	6 (16%)	5 (26%)	7 (32%)	2 (5%)
*IDH1*	11 (30%) **	1 (5%)	0 (0%)	0 (0%)
*EGFRvIII*	1 (3%)	0 (0%)	5 (23%)	1 (3%)
*PDGFRA*	4 (11%)	0 (0%)	0 (0%)	0 (0%)

An asterisk indicates *p*-values significant at 0.1 level, double asterisks indicate *p*-values significant at 0.01 level [6].
